# An Unusual Presentation of Delusional Companion Syndrome: A Case Report and Review of the Literature

**DOI:** 10.7759/cureus.51007

**Published:** 2023-12-23

**Authors:** Sabina Goldstein, David Jooryabi, Katherine Ammon, Joshua Delaney, Alejandro Rodulfo

**Affiliations:** 1 Psychiatry, Memorial Healthcare System, Hollywood, USA; 2 Psychiatry, Florida Atlantic University Charles E. Schmidt College of Medicine, Boca Raton, USA

**Keywords:** reproductive-psychiatry, antipsychotics, schizophrenia, delusional-companion-syndrome, delusional-misidentification-syndrome

## Abstract

Delusional companion syndrome, an uncommon subtype of delusional misidentification syndrome, has no prior reported cases in patients with primary psychotic disorders. We report a case of delusional companion syndrome in the absence of any organic brain disease, stroke, or severe brain injury, in a young female with schizophrenia. The patient is a 29-year-old G3P3 female, with a history of schizophrenia and major depressive disorder, who, after recently losing custody of her children, presented to the pediatric emergency department for evaluation of her baby doll, which she believed to be her child because the doll wasn’t eating or moving well. She became acutely agitated when providers declined to insert an IV line to hydrate the doll and required emergency treatment orders to de-escalate. The patient was admitted to the inpatient psychiatric unit and treated with oral aripiprazole 10 mg, before transitioning to her previous treatment of aripiprazole lauroxil at an increased dose of 882 mg monthly. By the end of admission, the patient grasped that the doll was a toy that had never been alive. This case demonstrates how delusional companion syndrome can occur in young patients with primary psychotic disorders, without a causative neurological insult, and can be treated with antipsychotics. More studies are necessary to further explore the relationship between primary psychotic disorders and delusional companion syndrome.

## Introduction

Delusional misidentification syndromes (DMS) are a family of complex delusional phenomena with distressing clinical presentations, often seen in association with psychiatric disorders as well as major neurocognitive disorders due to Lewy body or Alzheimer’s dementia [1]. The most well-studied DMS include Fregoli syndrome, intermetamorphosis, subjective doubles, paramnesia, and delusional companions [1]. These syndromes have also been observed in patients who have severe closed head traumas as well as vascular lesions of the brain. They are unique in their monothematic selectivity, only limited to a few people, places, or objects [2]. One hypothesis regarding the pathophysiology of these syndromes is that they may be caused by a disruption of the communication between multimodal cortical association areas as well as paralimbic and limbic structures, resulting in patients perceiving the stimuli of interest but not understanding its significance or relevance to self [2].

A less encountered subtype of DMS is delusional companion syndrome (DCS). Patients with DCS believe nonliving objects possess the ability to feel emotion, act and think independently, and possess consciousness [1]. Unlike other DMS, DCS has almost exclusively been seen in patients with Alzheimer's dementia, stroke, or traumatic brain injury [3]. We present a unique case of a 29-year-old female with a history of schizophrenia and major depressive disorder who presents with DCS in the absence of any organic brain disease, stroke, or severe injury.

## Case presentation

**Figure 1:**
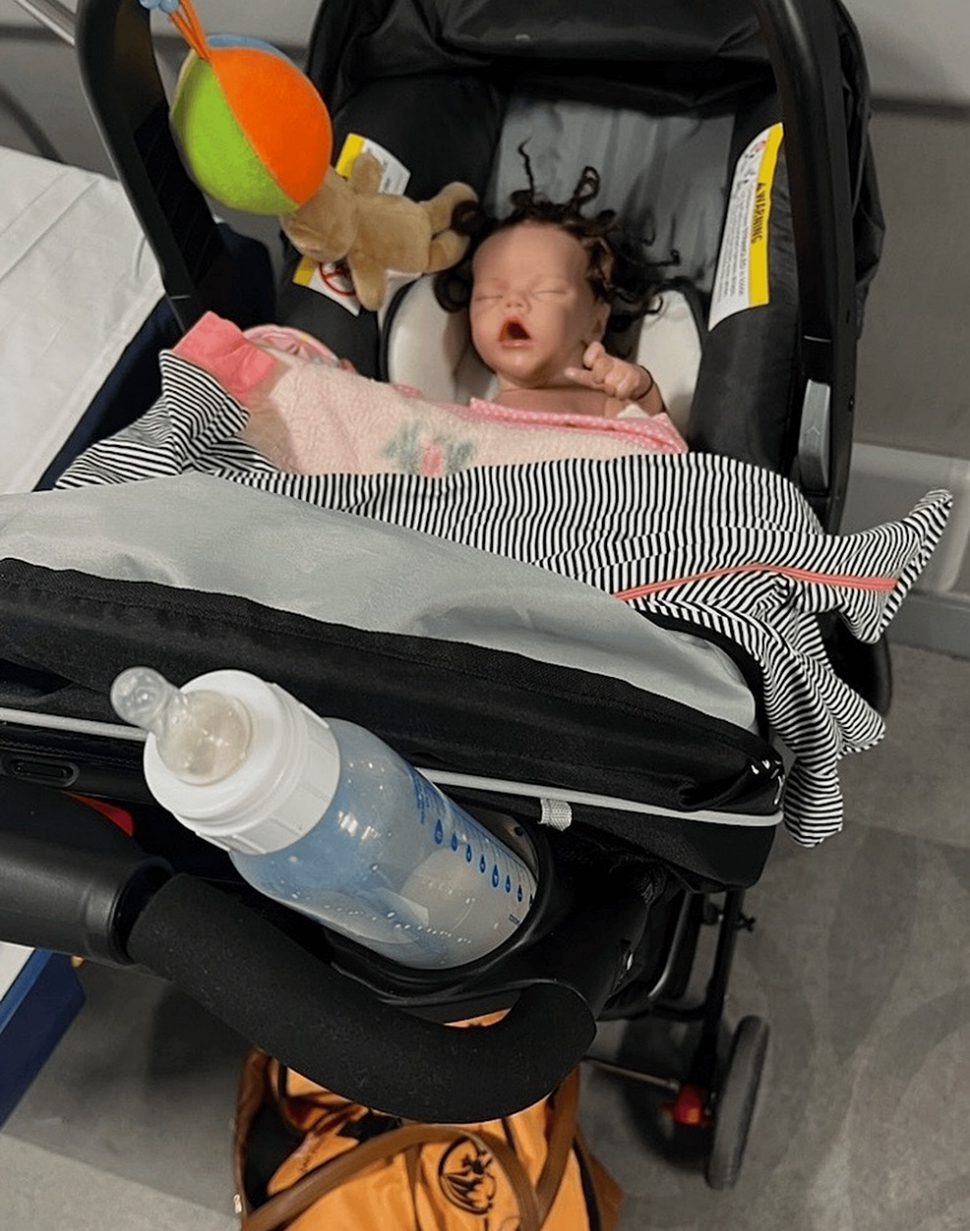
Patient’s “baby” in a stroller, hair braided Also visible: onesie, toys, blanket, bottle filled with formula, diaper bag

A 29-year-old G3P3 female with a history of schizophrenia and major depressive disorder, who recently lost custody of her children, was brought to the adult emergency department (ED) by nursing staff from the pediatric ED for a psychiatric evaluation. She initially presented to the pediatric ED requesting an evaluation for her infant daughter, whom she felt had not been eating or moving sufficiently and whom she had seen turning blue. When the staff looked in the stroller, they saw that the baby was a doll (Figure 1). Throughout the evaluation, the patient was only focused on her “daughter,” whom she believed was a living human infant. She repeatedly demanded that her baby get an intravenous line for hydration, and when staff refused, she became agitated, requiring an intramuscular injection of haloperidol 5 mg, lorazepam 2 mg, and diphenhydramine 50 mg while in the ED.

In an interview, the patient reported feeling depressed over the previous month because she missed her children (ages 1.5, 5, and 8), whom she lost custody of due to her inability to care for them. The patient lived at home with her mother and sister. The patient acknowledged that her doll, which she obtained two weeks prior, was purchased from a store and was delivered to her doorstep in a box. She reported feeding the doll with milk, watching the doll wet itself, and feeling the doll’s urine on her leg. She was concerned that her baby was choking because, recently, milk had been spilling out of the baby’s mouth and the baby was turning blue. Other presenting symptoms of the patient included thought blocking, constricted affect, and disorganized thought processes. The patient denied auditory and visual hallucinations, but her mother reported that she had seemed to be responding to internal stimuli at home. The patient had never had these specific delusions before.

The patient’s psychotic disorder had been controlled on monthly injections of aripiprazole lauroxil 662 mg for the preceding four years, but the patient had been nonadherent for the past five months. Psychiatric history was relevant for multiple psychiatric hospitalizations and involuntary holds due to psychosis in the setting of medication non-adherence, as well as a reported history of major depressive disorder episodes. She was psychiatrically hospitalized for the first time at the age of 22, due to psychosis, after she was brought in by her family with paranoid delusions, features of catatonia, and episodes of agitation, with no insight into her symptoms; she was re-hospitalized several months later due to bizarre behavior and auditory hallucinations and was tried on paliperidone palmitate long-acting injection at the time, before switching to aripiprazole lauxoril as an outpatient. The patient did not have a history of suicide attempts or suicidality, nor any family history of psychiatric illness. Based on collateral information from her family, it was unclear if her previously reported symptoms of depression (depressed mood, decreased motivation, poor sleep and appetite, decreased concentration, and psychomotor retardation) happened in the absence of psychosis or major psychosocial stressors. Schizophrenia was considered higher in the differential diagnosis, over major depressive disorder with psychotic features based on previous history and current presentation. Regarding social history, the patient had previously been physically abused by the father of her children while pregnant, and her children are currently living with their father. She was recently working for a fast food chain restaurant but lost her job during this most recent psychiatric decompensation.

Subsequent workup showed a comprehensive metabolic profile (CMP) and complete blood count (CBC) with differentials within normal limits, along with a negative beta-human chorionic gonadotropin (HCG). The urine drug screen was negative as well, just as it was on all of her prior hospitalizations at our hospital for the preceding five years. The most recent CT of the brain, performed less than a year prior, did not show any gray matter changes or chronic infarctions of the brain, as well as no major atrophy.

The patient was initiated on oral aripiprazole and then transitioned back to the long-acting injectable lauroxil formulation, this time at an increased dose of 882 mg monthly. The rationale for the increased dose was the issue of adherence and risk for a repeat hospitalization; this higher dose of the medication (882 mg) remains in the system longer than the 662 mg dose, and can even be dosed every six weeks instead of every four weeks as per the medication's package insert [4]. Throughout most of her hospitalization, the patient continued to believe that her doll was alive, becoming distressed when this delusion was explored. However, near discharge, she demonstrated partially improved insight, as she was able to explain that the doll was not ever alive and was actually a “silicone reborn real-life baby doll” she had purchased online. She expressed understanding that her doll would not require further hospital care. A picture of the doll, which was obtained with the patient's consent, is presented in Figure 1.

## Discussion

DMS are difficult to diagnose given their variety of presentations. Although there is now more information regarding these syndromes, their exact prevalence is still unknown, especially for DCS. There are currently no published case reports documenting DCS secondary to a primary psychiatric disorder, including schizophrenia.

There was a documented case of an 80-year-old patient with moderate Alzheimer's dementia who presented with DCS, believing that his toy doll was a living being [5]. He then was offered multiple other dolls on examination and believed all of them were also living beings, claiming he recognized the majority of them. A second case report documented a 14-year-old patient with autism spectrum disorder who believed he had multiple imaginary friends whom he was in close communication with; however, this did not involve any physical entity or misidentification, which is inconsistent with DMS [6].

Currently, it is believed that DMS are multifactorial in etiology, involving dysregulation of paralimbic and limbic structures as well as the uncinate fasciculus fiber tract, resulting in the ability for patients to perceive the stimuli of interest but not its significance or relevance to self [2,7]. Right parietal lobe and temporal lobe dysfunction have been implicated in DMS as well, with one case series reporting marked dysfunction in regional right cerebral brain flow in three patients presenting with DMS [4]. In an additional study of 10 post-stroke patients with DMS, all 10 patients had lesions in their right hemispheres, including the insula, inferior frontal lobe, anterior temporal lobe, and subcortical limbic system. These areas share a connection via the uncinate fasciculus suggesting that dysfunction of this area is implicated in DMS [8]. It is not known whether DCS shares the same pathophysiology as DMS.

Current management for DMS involves treating the underlying psychiatric disorder with antipsychotics and/or antidepressants depending on the primary diagnosis. Group therapy has also been proposed [1]. Given the paucity of information regarding the treatment of DCS specifically, our patient was treated with oral aripiprazole and then the long-acting formulation, given her prior positive response to this agent and her previous documented history of schizophrenia, a diagnosis we agreed with based on her presentation. This treatment led to symptom regression and improved insight. This provides some evidence that antipsychotics may provide symptomatic improvement of DCS in the context of a primary psychotic disorder.

## Conclusions

DMS is a family of complex delusional phenomena with varying clinical presentations, often seen in association with psychiatric disorders as well as major neurocognitive disorders. DCS is a rare subtype of DMS that has previously only been reported in the literature in the context of patients with Alzheimer’s dementia. In this case, a patient with a history of schizophrenia was found to have DCS and benefited from treatment with a second-generation antipsychotic. Further research is required to elucidate the prevalence, pathophysiology, and optimal treatment for DCS.
